# Automatic detection of pain using machine learning

**DOI:** 10.3389/fpain.2022.1044518

**Published:** 2022-11-10

**Authors:** Brent D. Winslow, Rebecca Kwasinski, Kyle Whirlow, Emily Mills, Jeffrey Hullfish, Meredith Carroll

**Affiliations:** ^1^ Design Interactive, Inc., Orlando, FL, United States; ^2^ Arcanium Software, LLC, Tampa, FL, United States; ^3^ ATLAS Lab, College of Aeronautics, Florida Institute of Technology, Melbourne, FL, United States

**Keywords:** pain, classification, machine learning, heart rate variability, wearable devices

## Abstract

Pain is one of the most common symptoms reported by individuals presenting to hospitals and clinics and is associated with significant disability and economic impacts; however, the ability to quantify and monitor pain is modest and typically accomplished through subjective self-report. Since pain is associated with stereotypical physiological alterations, there is potential for non-invasive, objective pain measurements through biosensors coupled with machine learning algorithms. In the current study, a physiological dataset associated with acute pain induction in healthy adults was leveraged to develop an algorithm capable of detecting pain in real-time and in natural field environments. Forty-one human subjects were exposed to acute pain through the cold pressor test while being monitored using electrocardiography. A series of respiratory and heart rate variability features in the time, frequency, and nonlinear domains were calculated and used to develop logistic regression classifiers of pain for two scenarios: (1) laboratory/clinical use with an F1 score of 81.9% and (2) field/ambulatory use with an F1 score of 79.4%. The resulting pain algorithms could be leveraged to quantify acute pain using data from a range of sources, such as ECG data in clinical settings or pulse plethysmography data in a growing number of consumer wearables. Given the high prevalence of pain worldwide and the lack of objective methods to quantify it, this approach has the potential to identify and better mitigate individual pain.

## Introduction

Pain is one of the most common symptoms reported by individuals presenting to hospitals and clinics (1) and is defined as an unpleasant sensory and emotional experience induced by noxious stimuli detected by nociceptive neurons (2). The economic cost of pain including medical care, lost wages, and reduced productivity eclipses heart disease, cancer, and diabetes (3). The concurrent opioid and COVID-19 epidemics have accelerated the costs associated with pain along with an increasing number of overdoses (4). Previous research has defined the underlying mechanisms associated with nociceptive pain and distinguished two types, including first pain, and secondary/affective pain (5). First pain is associated with myelinated A-Delta fibers which synapse onto primary somatosensory cortex, and provide fast localization of injury, triggering withdrawal reactions. Secondary pain is associated with the activation of slower, unmyelinated C-fibers which synapse onto the forebrain/affective areas, providing the emotional context to pain. Pain can also be characterized by the duration or time course; while acute pain allows for rapid localization of injury, prevention of self-harm, and generally persists while tissues heal, up to one third of individuals in the United States experience chronic pain and associated disability associated with diseases such as cancer, post-surgical and post-traumatic pain, neuropathic pain, headache, visceral, and musculoskeletal pain (6). Given its significant economic and social impacts, the ability to quantify and treat pain is of high importance.

Pain is commonly assessed through self-report (7), but such approaches are highly variable, subjective, and are influenced by a number of internal and external factors (8). For example, recent reports indicate that common self-reported pain scales used for screening only have modest accuracy (7), and some patients will systematically under or over-report pain (9, 10). Given the risks of relying on self-reported pain, other groups have attempted to leverage clinical imaging such as fMRI to analyze brain regions associated with pain, which have revealed a pain matrix of regions reliably activated by painful stimuli (11). These regions include the somatosensory cortex which localizes pain, the anterior cingulate cortex and insula, which are associated with the emotional and motivational aspects of pain (12), along with higher brain areas. While such an approach has provided for the development of high specificity/high sensitivity biomarkers for pain (13), it still requires the use of expensive imaging equipment and highly trained personnel. There is thus a need for an objective approach to pain identification that is both cost-effective and scalable to minimize the reliance on costly and limited neuroimaging.

Available evidence suggests that pain has objective, physiological signatures that manifest outside of the brain. For example, acute and chronic pain are associated with stereotypical physiological alterations (14) such as increasing cardiovascular activity including heart rate (HR), blood pressure and heart rate variability (HRV) (14–17), respiration rate and depth (18), and electrodermal activity (19, 20). Such observations form the scientific basis for current guidelines for patient care in anesthesiology (21), but also have the potential to objectively assess pain in ambulatory settings. Combined with advances in non-invasive persistent physiological monitoring devices, physiological pain assessment may allow for real-time, objective pain sensing and mitigation. In the current study, a physiological dataset associated with acute pain induction in healthy adults was leveraged to develop algorithms capable of detecting pain in real-time. These methodologies provide a foundation for future work in developing advanced pain classification algorithms to be used in field/ambulatory environments using wearable sensors.

## Methods

### Participants

All methods involving human subjects were approved by an independent Institutional Review Board (Copernicus Group, Durham, NC). Forty-one participants [thirty-eight male; average age 21.8 ± 2.4 (SD) years] completed and received payment of $100 USD for participation in the study. The demographics were designed to correlate with the United States military service member population. All participants were recruited from the community and met minimum requirements including age (18–30), normal visual acuity, and no medical conditions such as endocrine disorders.

### Data collection procedure

Upon arrival, participants provided written informed consent and completed a demographics questionnaire. Wireless physiological sensors were then placed on the participants, followed by a 5-min recording of baseline (BL) physiological activity while participants remained seated. Participants then underwent the cold pressor test (CPT), consisting of up to 3 min of non-dominant hand immersion in ice cold water (1–2°C) under experimenter observation. The CPT was initially developed in 1932 as a clinical cardiovascular challenge to monitor changes to HR and blood pressure (17) and is considered a reliable experimental method for controlled pain induction (22). Physiological measures (detailed in Section “Physiological measurements”) were captured throughout the baseline and pain-induction phases. At the end of the experiment, participants were debriefed and paid for their participation.

### Physiological measurements

Participants were fitted with a 3-lead electrocardiogram (ECG) with bandlimits set between 1 and 35 Hz. ECG was sampled at 500 Hz and wirelessly sent to an MP-150 system running AcqKnowledge software (Biopac Systems, Goleta CA). Gain was set on the ECG channel to 2000.

### Data featurization

**Figure 1:**
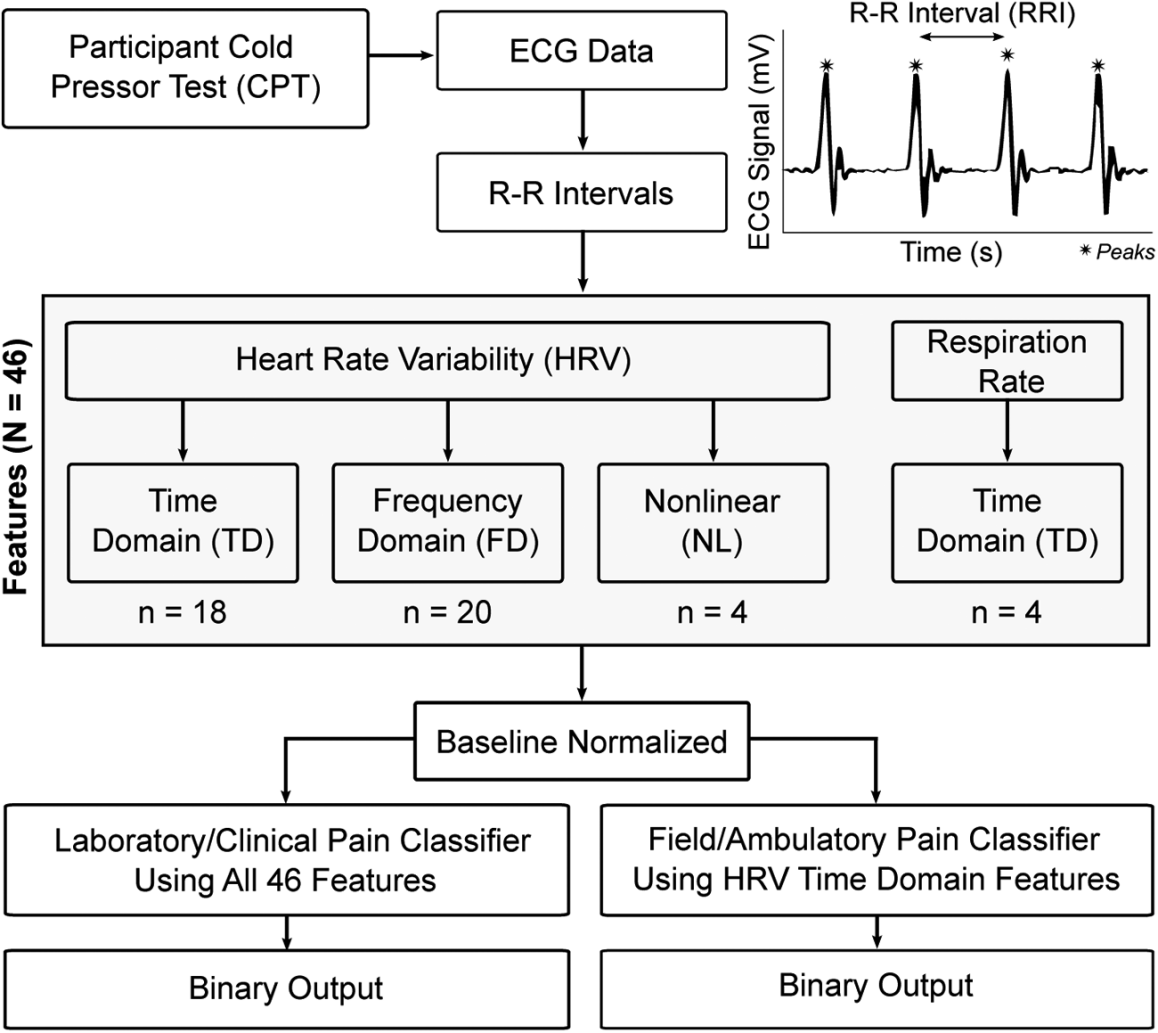
Data measurement, featurization, and modeling overview; *peaks of the ECG signal.

Peak detection was used to identify and extract the R-R intervals (RRI) for each ECG recording (Figure 1). For each recording, the RRIs were separated into 60-second, non-overlapping epochs to coordinate with known temporal dynamics of pain (23). This RRI data was analyzed to convert the time-series data into featurized observations for use as model inputs (Figure 1). These features (*N* = 46) fell into one of four categories: (1) time domain HRV, *n* = 18; (2) frequency domain HRV, *n* = 20; and (3) nonlinear HRV, *n* = 4; and (4) respiration, *n* = 4 which are described in the following sections.

HRV features were calculated using the pyHRV open source toolbox (24). The toolbox bundles a selection of functions to compute time domain, frequency domain, and nonlinear HRV features. Time domain features contain statistical information from two different classes: (a) features derived directly from RRI or instantaneous HR, and (b) those derived from the differences between successive RRI. Time domain features included RRI (min, max, mean, count), differences in successive RRI (RRIdiff; mean, min, max), heart rate (HR; mean, min, max, standard deviation), standard deviation of RRI (SDNN), root mean square of RRI (RMSSD), standard deviation of successive RRIdiff (SDSD), number of RRI greater than 50 ms (RRI-50), ratio between RRI-50 and total number of RRI (pRRI-50), number of RRIdiff greater than 20 ms (RRI-20), and the ratio between RRI-20 and the total number of RRI (pRRI-20).

Frequency domain HRV was obtained using Fast Fourier Transform to compute the power spectral density, followed by an analysis of very low frequency (VLF) power (0–0.04 Hz), low frequency (LF) power (0.04–0.15 Hz), indicative of sympathetic activity, high frequency (HF) power (0.15–0.4 Hz), indicative of vagal activity, and very high frequency (VHF) power (0.4–3 Hz) (25). For each frequency band, peak frequency, absolute power, log power, and relative power were calculated. Normalized power was also calculated for LF and HF, along with LF/HF ratio and total power.

Nonlinear HRV parameters were also included to enhance the unpredictability of the R-R series caused by various complex physiological dynamics of the cardiovascular system that lead to HRV (26, 27). The Poincaré plot is a scatter plot where a given RRI is plotted against its successor RRI, which allows a rapid first judgment of a subject's health as the shape of the scatter plot provides a visual representation of the overall HRV (28). In addition to the plot, analysis of the Poincaré ellipse provides additional parameters that can be used for an analysis of the RRI scatter plot: standard deviation of the minor axis (SD1), standard deviation of the major axis (SD2), SD1/SD2 ratio, and the ellipse area.

Respiration was derived from RRI data by leveraging the respiratory sinus arrhythmia (RSA). RSA represents HRV in synchrony with respiration, by which the RRI on an ECG is shortened during inspiration and prolonged during expiration (29). First, the RRIs were linearly interpolated to create a uniformly sampled time-series (f_s_ = 4 Hz). This interpolated time-series data was then filtered using a Butterworth bandpass filter in the range of 0.2–0.8 Hz to isolate the frequency components relevant for respiration. Then, a peak detection algorithm was run on the bandpass-filtered signal to detect breaths. The inter-breath intervals (IBI) were then calculated (in ms) for each of the detected peaks/breaths. Finally, the IBIs were converted to respiration rate (in breaths per minute). The respiratory features used to create the pain classifier were calculated based on the instantaneous respiration rate data. These included a total of four time domain features (mean, min, max, standard deviation) in breaths/min.

After calculating all features (*N* = 46) for each epoch, the features were baseline normalized. This was done to reduce the confounding influence of interindividual variability on the classifier. This normalization process began by pairing each participant's BL epochs with all of their other epochs. The features were then subtracted between each of these BL/BL and BL/CPT epoch pairs, and the absolute values of the differences were taken. The resulting data that were used as classifier inputs therefore describe the magnitude of the difference between an epoch of data and a physiological baseline for a given participant.

### Data modeling

To ensure that a pain classification approach was developed that could be used outside of controlled environments, two pain classification pipelines were generated: (1) laboratory grade classifier, which is more computationally intensive and intended to be used on higher-end computing equipment; and (2) field grade classifier leveraging only time-domain HRV features and intended to be used with current wearable sensors (Figure 1). RRIs were used for derivation of all features and initial model selection of the field grade classifier in an effort to mimic a real-world collection scenario from a smartwatch.

Training and test datasets were created dynamically using the Leave-One-Subject-Out (LOSO) cross-validation method. LOSO was utilized in evaluation to allow for better subject-to-subject variation in training while also limiting autocorrelation for a single subject. Next, a BL vs. CPT preprocessing and classification pipeline was developed utilizing Python's Scikit-learn (sklearn) library (30). The pipeline applied a standard scaler (i.e., subtract mean and divide by standard deviation) to all features as well as a principal component analysis (PCA) for dimensionality reduction. Finally, a logistic regression classifier was implemented to differentiate BL vs. CPT.

### Data analysis and statistics

For each classifier developed, several classification metrics were calculated using the sklearn library including precision, recall, support, and F_1_ score. Precision is defined as the ratio of true positives to the sum of true and false positives. Recall is defined as the ratio of true positives to the sum of true positives and false negatives. The F_1_ score is the weighted harmonic mean of precision and recall with a maximum value of 1.0 and a minimum value of 0.0. Support is the number of occurrences of the given class in the overall dataset.

## Results

The sociodemographic characteristics of the participants in the study are presented in Table 1. Participant ages ranged from 19 to 30, with a mean of 21.9 ± 2.4 (SD) years. The average duration of the CPT was 2.51 (0.80) min, with 29/41 participants completing the full 3-min duration. Among the 12 individuals who did not complete the CPT, the average duration was 1.34 (0.44) min.

**Table 1:** List of sociodemographic factors of study sample (*N* = 41).

	Study sample % (*n*)
Gender	
Male	92.7 (38)
Female	7.3 (3)
Age group	
18–21	51.2 (21)
22–25	39.0 (16)
26–30	9.8 (4)
Education	
High School Diploma	26.8 (11)
Some College/University	51.2 (21)
University Degree	22.0 (9)

### Feature permutation importance

**Figure 2:**
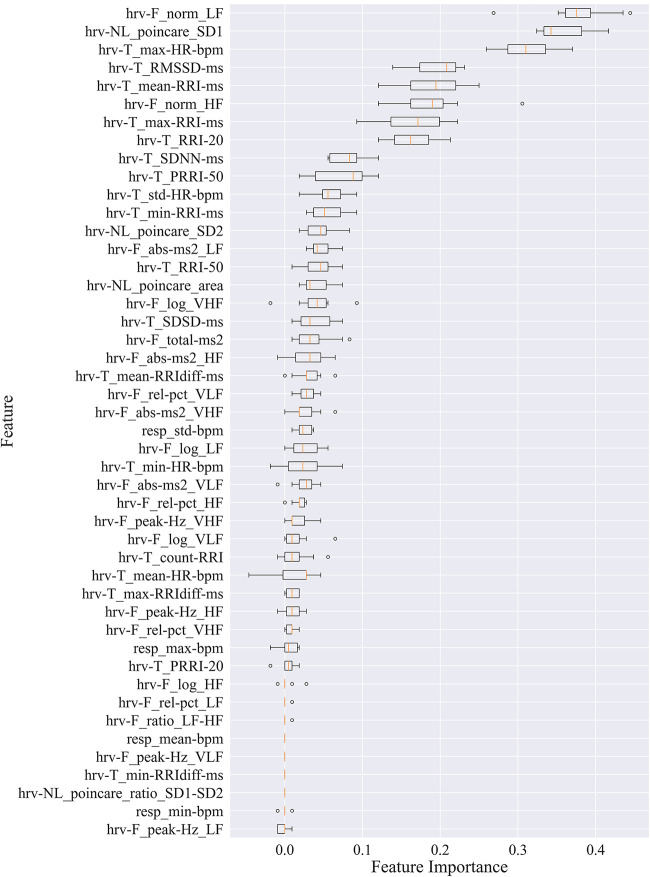
Permutation importance utilized to estimate individual feature importance. Boxplots for each feature are plotted by importance level, with median shown as vertical lines, standard deviation bars, and outliers shown as small circles. LF, low frequency; HF, high frequency; VLF, very low frequency; VHF, very high frequency; P, power; Rel, relative; Abs, absolute; SD1, standard deviation of minor axis; SD2, standard deviation of major axis; RRI, R-R interval; RRIdiff, differences in successive RRI; RMSSD, root mean square of RRI; SDNN, standard deviation of RRI; SDSD, standard deviation of successive RRIdiff; RRI-20, number of RRIdiff greater than 20 ms; RRI-50, number of RRI greater than 50 ms; pRRI-20, ratio between RRI-20 and total RRI; pRRI-50, ratio between RRI-50 and total RRI.

Box plots in Figure 2 show the feature importance values across iterations of the algorithm for all 41 participants. These values are used to compute the average feature importance and the corresponding standard deviations shown in the bar chart. The *x*-axis shows the impact that permuting a given feature had on the model's prediction score. The *y*-axis shows the different features in the relative importance order. The minimum, first quartile, median, third quartile, and a maximum of the feature importance values across different iterations of the algorithm are shown by each box.

**Figure 3:**
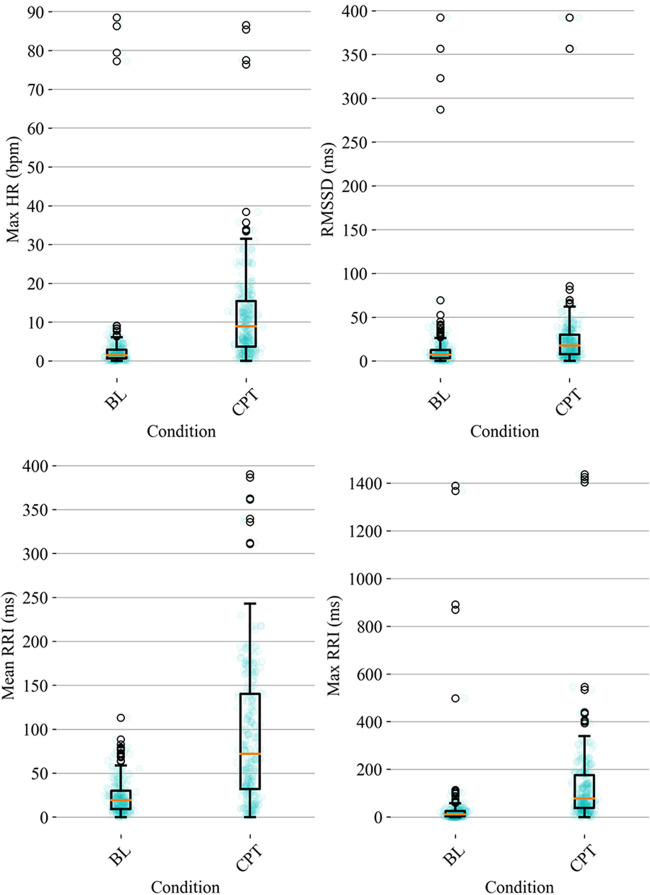
Baseline-normalized, HRV time domain features contributing most to the model included maximum HR, RMSSD, mean RRI, and max RRI. The data overlay on each boxplot represents the entire dataset (blue circles) used to generate the corresponding boxplot with medians shown in red and outliers shown as black circles.

**Figure 4:**
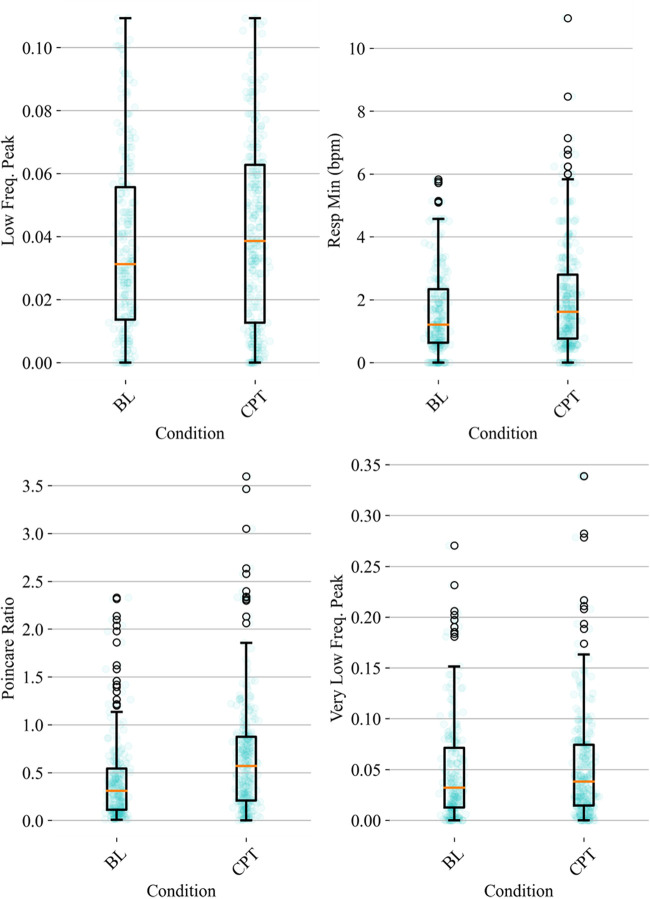
Baseline-normalized, non-HRV time domain features contributing least to the model included low frequency (LF) peak, the minimum respiration rate, Poincare ratio, and very low frequency (VLF) peak. The data overlay on each boxplot represents the entire dataset (blue circles) used to generate the corresponding boxplot with medians shown in red and outliers shown as black circles.

HRV time domain features most heavily influenced the model F1 score. To further illustrate the importance of these features in classifying pain, Figures 3, 4 show boxplots to visualize differences in features for the BL and CPT (pain) conditions. The top four contributing HRV time domain features included the maximum HR, mean and max RRI and RMSSD. Figure 3 shows clear differences within these top features across the BL and CPT classes. Conversely, the lowest contributing HRV features included low frequency peak, the minimum respiration rate, Poincare ratio, and VLF frequency peak. Figure 4 shows little distinction within these lowest four features across the BL and CPT classes.

### Laboratory/clinical classifier

The computationally intensive, laboratory/clinical pain classifier was generated utilizing all available features (*N* = 46, listed in Figure 2). This laboratory-grade approach classified pain at an F1 level of 81.9% (Table 2).

**Table 2:** Sklearn classification report for laboratory grade pain classifier with all features.

	Precision	Recall	F_1_-score	Support
BL	0.765	0.861	0.811	231
CPT	0.875	0.785	0.828	284

BL, baseline; CPT, cold pressor test (pain induction task).

### Field/ambulatory classifier

Since HRV time-domain features had the highest influence on the model, the same preprocessing and classification pipeline described above was utilized to create a field grade classification model with only the HRV time-domain features (*n* = 18). This algorithm was capable of classifying pain at an F1 level of 79.4% (Table 3). Finding the optimal balance between model complexity and performance ensures both that the model can run efficiently when deployed and that the potential for overfitting is minimized.

**Table 3:** Sklearn classification report for field grade pain algorithm with HRV time domain features.

	Precision	Recall	F_1_-score	Support
BL	0.736	0.844	0.786	231
CPT	0.856	0.754	0.801	284

BL, baseline; CPT, cold pressor test (pain induction task).

## Discussion

The current study demonstrated the ability to create a series of algorithms capable of automatically detecting pain in controlled and natural environments. Two algorithms were developed, one that is more computationally intensive with a higher number of features and an F1 score of 81.9%, as well as a less computationally intensive classifier more suited for field settings with fewer features and an F1 score of 79.4%. Such approaches could be leveraged to quantify acute pain in the presence of RRI data from a range of sources, from ECG data in clinical settings to data derived from PPG in consumer wearables. The use of the CPT allowed for controlled, replicable pain induction promotes a moderately painful but rapidly reversible stimulus, as widely reported *via* self-report (31) or behavioral change (32). Given the high prevalence of pain worldwide, which reaches up to 25% of the population in some countries (33), and the lack of objective methods to classify pain, this approach has the potential to identify and better mitigate individual pain.

A number of previous groups reported physiology-based approaches to pain classification, but to date such approaches require the use of laboratory equipment or controlled settings (34), and have leveraged modest sample sizes. For instance, one group leveraged fMRI data and support vector machines to develop a pain algorithm with 81% accuracy in a group of 24 subjects (35). Another group reported an electroencephalography (EEG)-based pain algorithm by analyzing 64 channels of EEG data with an accuracy of 80% in a group of 29 subjects (36). Multimodal approaches integrating cardiovascular, respiratory, electrodermal, and electromyography sensors were able to classify acute heat pain at 76% accuracy in a group of 30 subjects but required significant experimental setup and was not realistic for field environments (37). Previous ECG-based methods developed for postoperative patients have reported accuracy levels from 62% to 84% in a group of 25 subjects, but were targeted only to clinical environments (38). Deep-learning approaches are expected to improve on this accuracy, at the cost of significant algorithm complexity and reduced transparency (39). Remarkably, the current study presented an 79.4% accurate classifier of acute pain leveraging only HRV features from sample size approximately 25% larger than previous work, allowing for field grade classification in natural environments.

Various approaches to HRV quantification have been used in the past to classify pain. A systematic review argued for the use of frequency domain measures, including increased low frequency (sympathetic) and decreased high frequency (vagal) HRV for monitoring acute pain (15). The authors recommended assessing individual differences and the impact of alterations on pain induction tasks to HRV. An additional systematic review focused on changes to HRV under chronic pain (40). Leveraging both time and frequency domain HRV metrics, the authors identified a moderate to large effect of decreased high-frequency HRV in chronic pain, indicative of decreased parasympathetic activity. Our data suggests that acute thermal pain induction, using cold stimuli can be assessed with high accuracy in field settings using time domain HRV. As various HRV changes have also been noted following psychological stress (41), movement (42), and mental workload (43), understanding context is critical to the successful use of limited sensor approaches to quantifying human status.

The current effort achieved comparable accuracy to previous approaches by leveraging RRI data which is becoming common in emerging wearable fitness devices. Such data can be obtained *via* monitoring changes to HR from optical sources including PPG, ECG recordings, or radio frequency at various body sites including the chest, wrist, or earlobe (44). Increasingly, the ability to gather cardiovascular data from non-traditional sources, such as cameras or smartphones, will allow for more persistent and accurate tracking. For instance, independent component analysis of visual spectrum imagery from the face, followed by Fast Fourier Transform within a biologically relevant frequency band was capable of extracting cardiac pulse with high accuracy from multiple subjects simultaneously in the presence of motion artifacts, differences in skin coloration, and illumination (45). In addition, digital phenotypes (46) developed using sensors embedded in smartphones and machine learning have shown the potential to extract cardiovascular variables without the need for external monitoring devices (47).

The average duration of the CPT was similar to previous studies (48), but the percentage of participants that did not complete the CPT was approximately double previously reported percentages (49). Duration of CPT has been used previously as a proxy for pain tolerance (32). In our study, participants found the CPT highly painful, unpleasant, difficult to complete, and stressful (49). Since pain experience is associated with significant individual differences, various demographic factors including age and ethnic group, along with genetic and psychosocial factors could contribute to the individual differences in pain toleration observed in this study (50). Additionally, physiological differences were seen in baseline-normalized responses to pain-induction tasks, reflected by the outlier datapoints observed in Figures 3, 4, and could be associated with underlying cardiovascular health (51), or pain tolerance (52). Sex-related influences on pain and analgesia have also been previously reported (53). However, such largely self-reported differences are not associated with discernible differences between sexes in cardiovascular activity under pain (16). Thus, although the sample population was majority male, the algorithms reported in this manuscript could be expected to perform well in both sexes. However, additional replication, expansion, and increasing the diversity of the underlying dataset is needed prior to generalizing to a larger cohort.

In summary, a high accuracy classification of acute pain using HRV time domain features was developed with potential to be implemented with existing and emerging tools in natural environments. Current work is focused on altering the binary output to reporting pain on a scale to more closely model existing pain reporting scales. Current work is also focused on leveraging this approach to quantify and mitigate pain in fighter pilots, who are at high risk for experiencing chronic pain in the upper back and neck due to exposure to high G-forces while flying (54). A mobile application that implements the automated pain classification algorithm along with flight approved, wireless wearable sensors are being leveraged to detect and mitigate pain.

## Data Availability

The raw data supporting the conclusions of this article will be made available by the authors, without undue reservation.
